# Supplementary material for: DNA Methylation Module Network-Based Prognosis and Molecular Typing of Cancer
Source: Genes (Basel). 2019 Jul 28;10(8):571. doi: 10.3390/genes10080571 (PMC6722866; doi:10.3390/genes10080571)
Supplement: Supplementary file 1 [file genes-10-00571-s001.pdf]

The file includes ten figures (Figure S1 – Figure S10) and four tables (Table S1- S4).

Figures

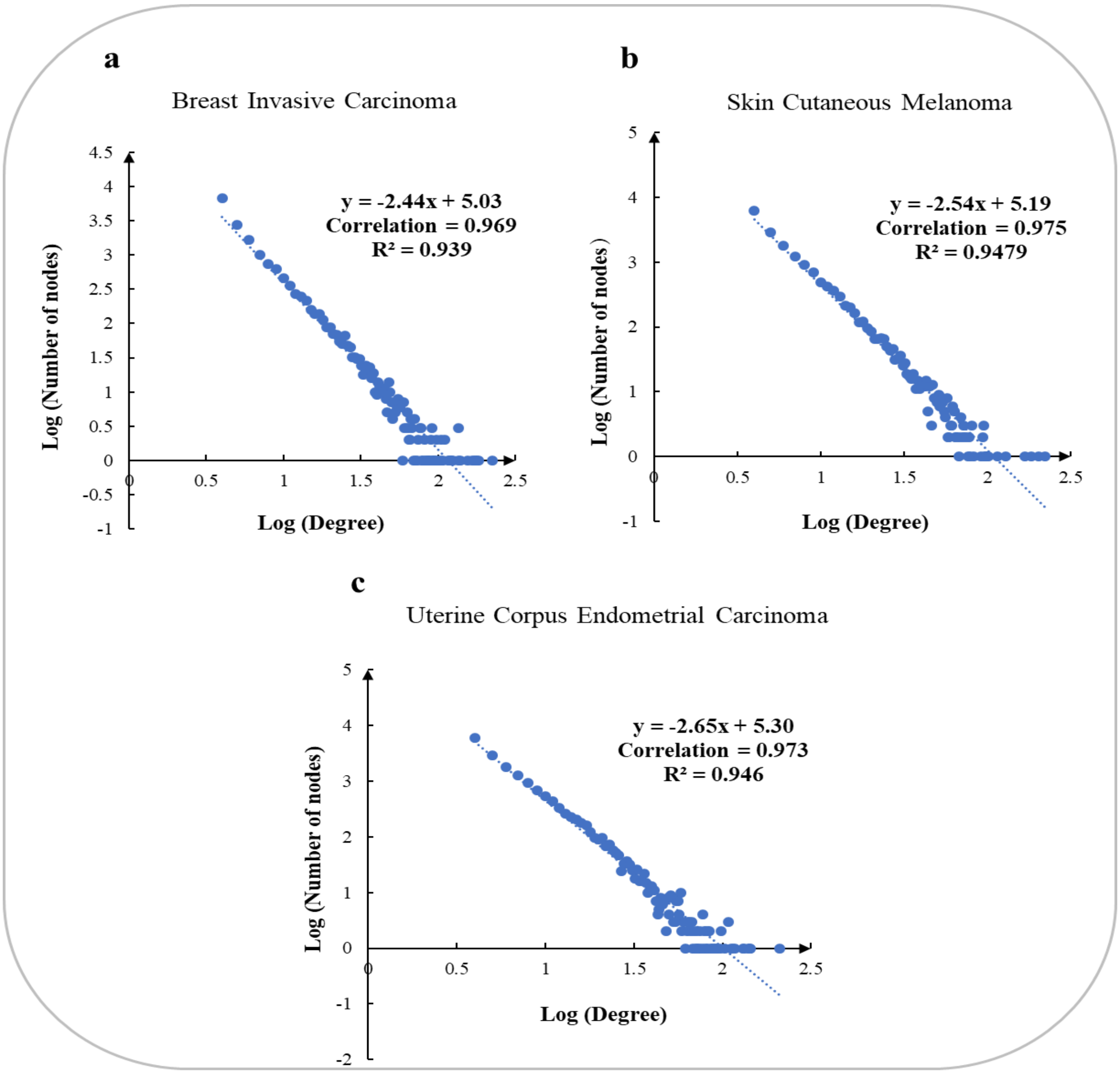

Figure S1. Power-law distribution fitting of nodal degrees in three cancer co-methylation networks. a. The fitting results of node degrees in breast invasive carcinoma co-methylation network; b. The fitting results of nodal degrees in skin cutaneous melanoma co-methylation network; c. The fitting results of nodal degrees in uterine corpus endometrial carcinoma co-methylation network

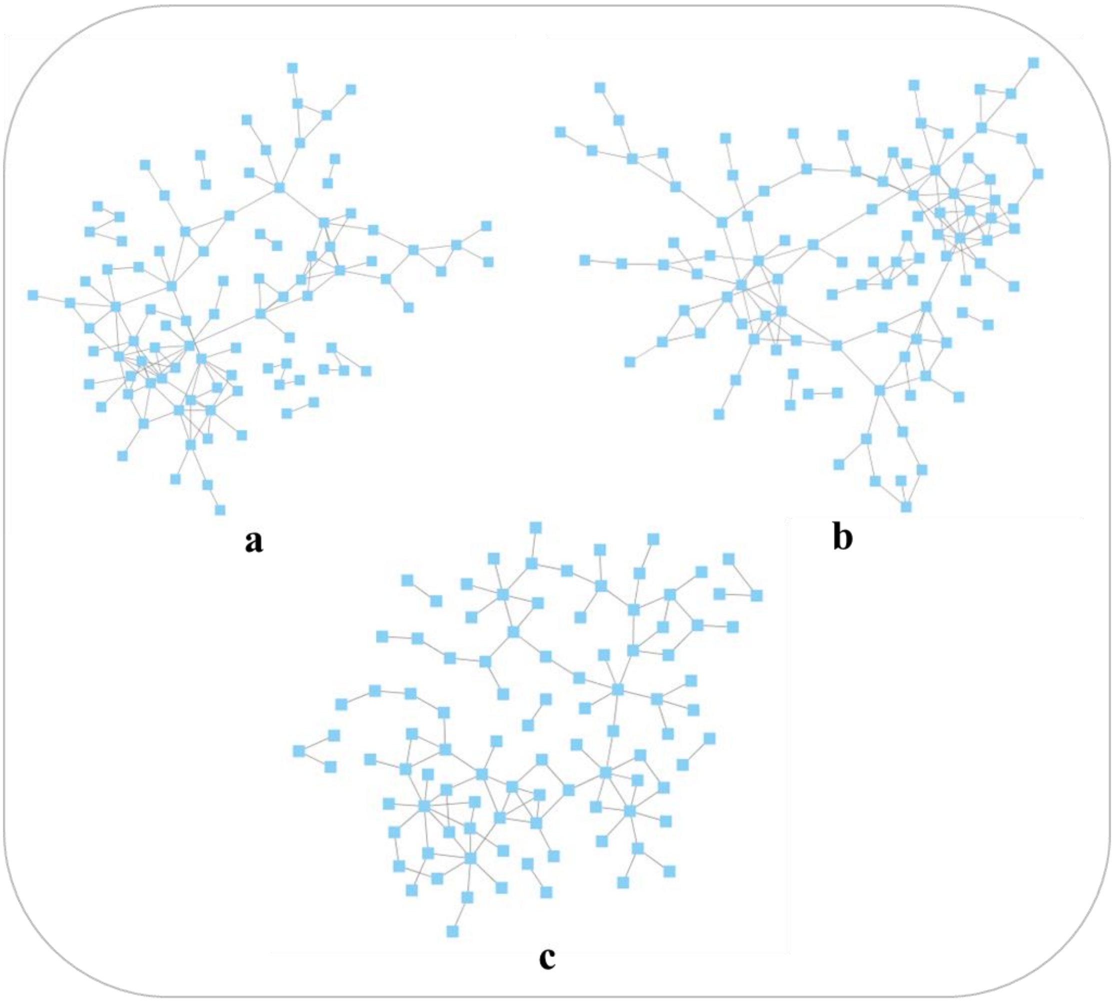

Figure S2. The module networks of three cancers. a. The module network of breast invasive carcinoma; b. The module network of skin cutaneous melanoma; c. The module network of uterine corpus endometrial carcinoma

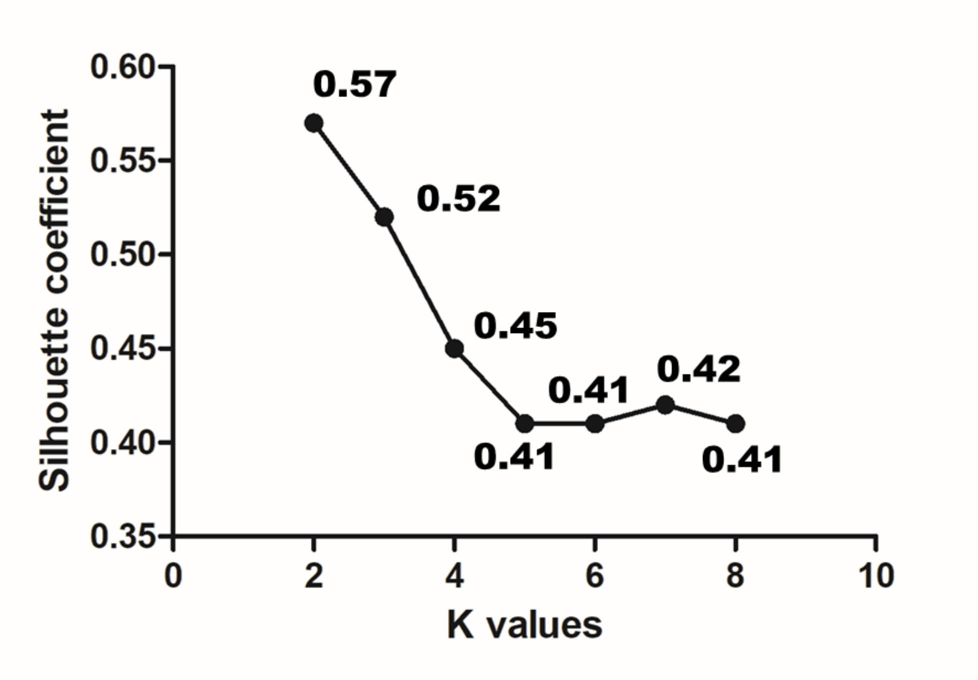

Figure S3. Silhouette coefficients of breast invasive carcinoma samples at different K values

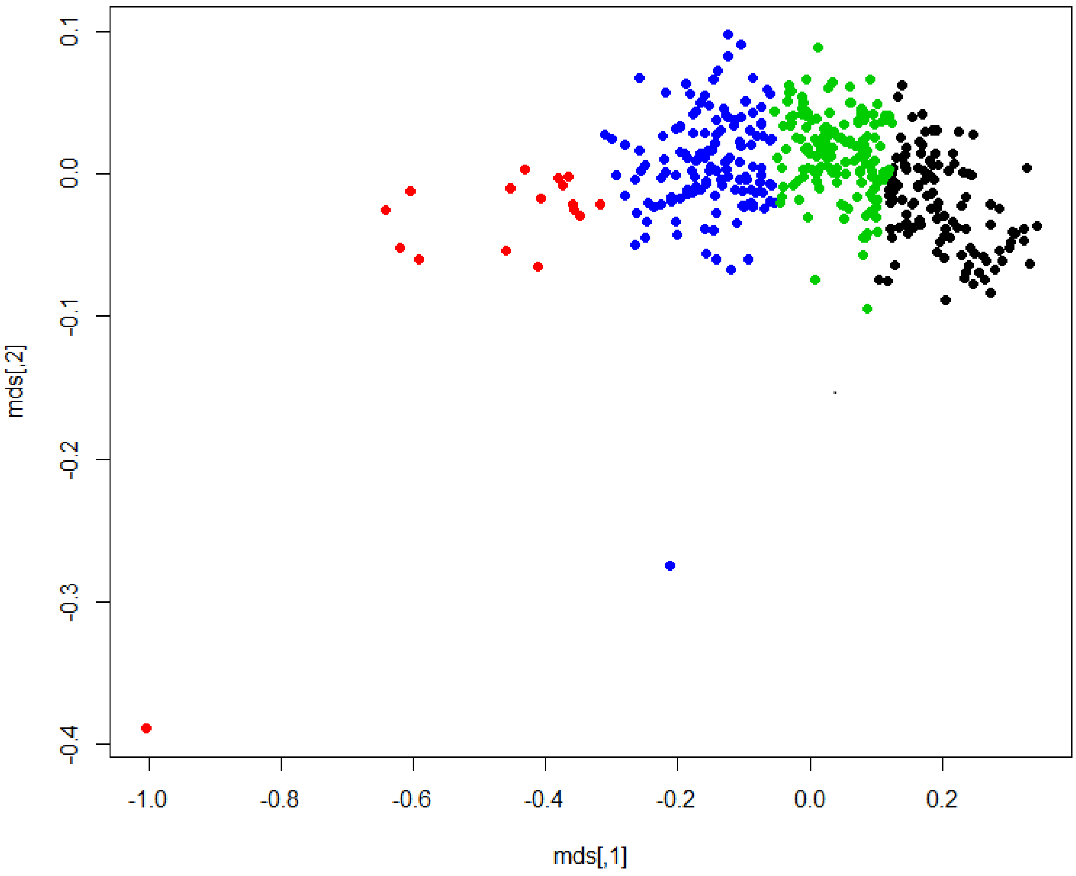

Figure S4. Clustering results of breast invasive carcinoma samples by K-means algorithm (K = 4, after MSD conversion)

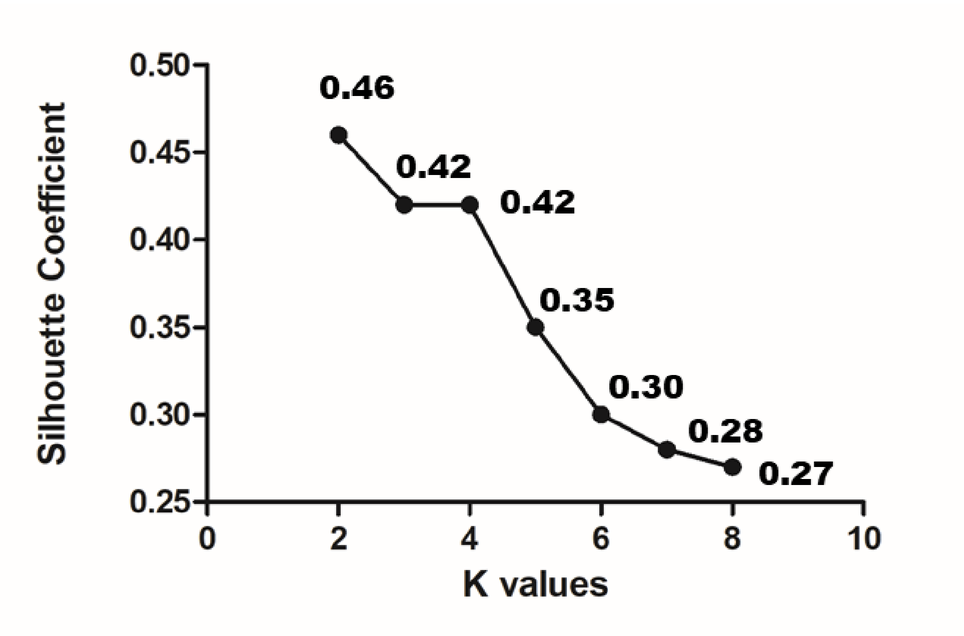

Figure S5. Silhouette coefficients of skin cutaneous melanoma samples at different K values

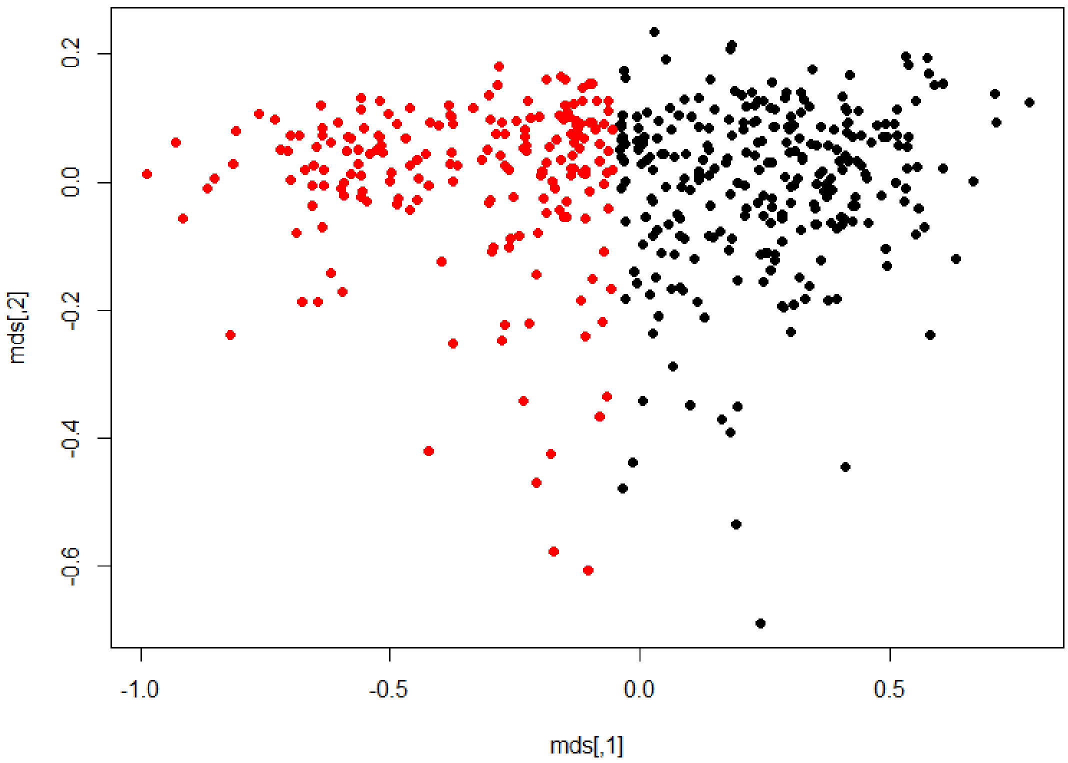

Figure S6. Clustering results of skin cutaneous melanoma samples by K-means algorithm (K = 2, after MSD conversion)

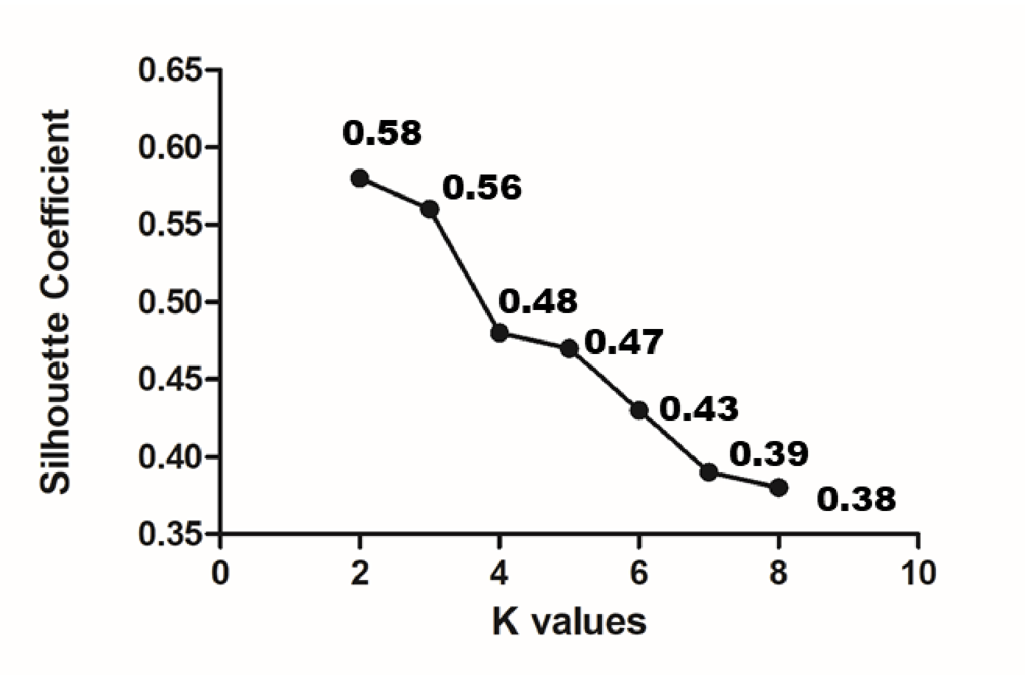

Figure S7. Silhouette coefficients of uterine corpus endometrial carcinoma samples at different K values

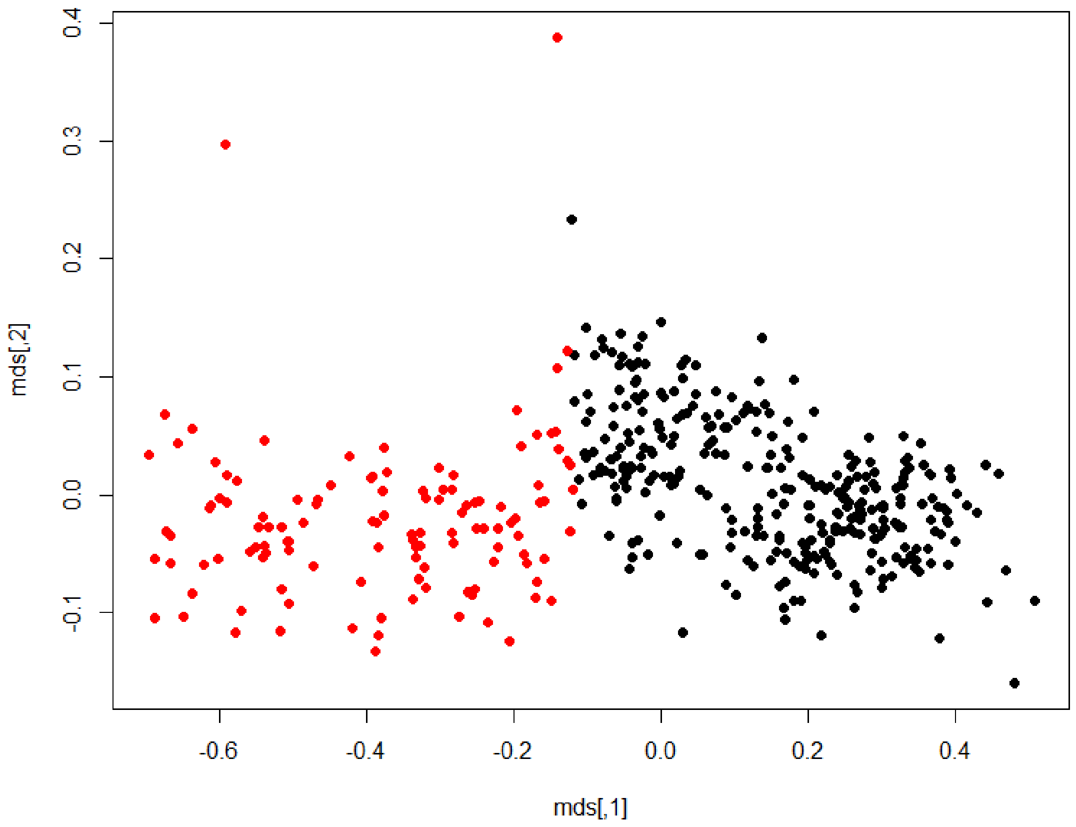

Figure S8. Clustering results of uterine corpus endometrial carcinoma samples by K-means algorithm (K = 2, after MSD conversion)

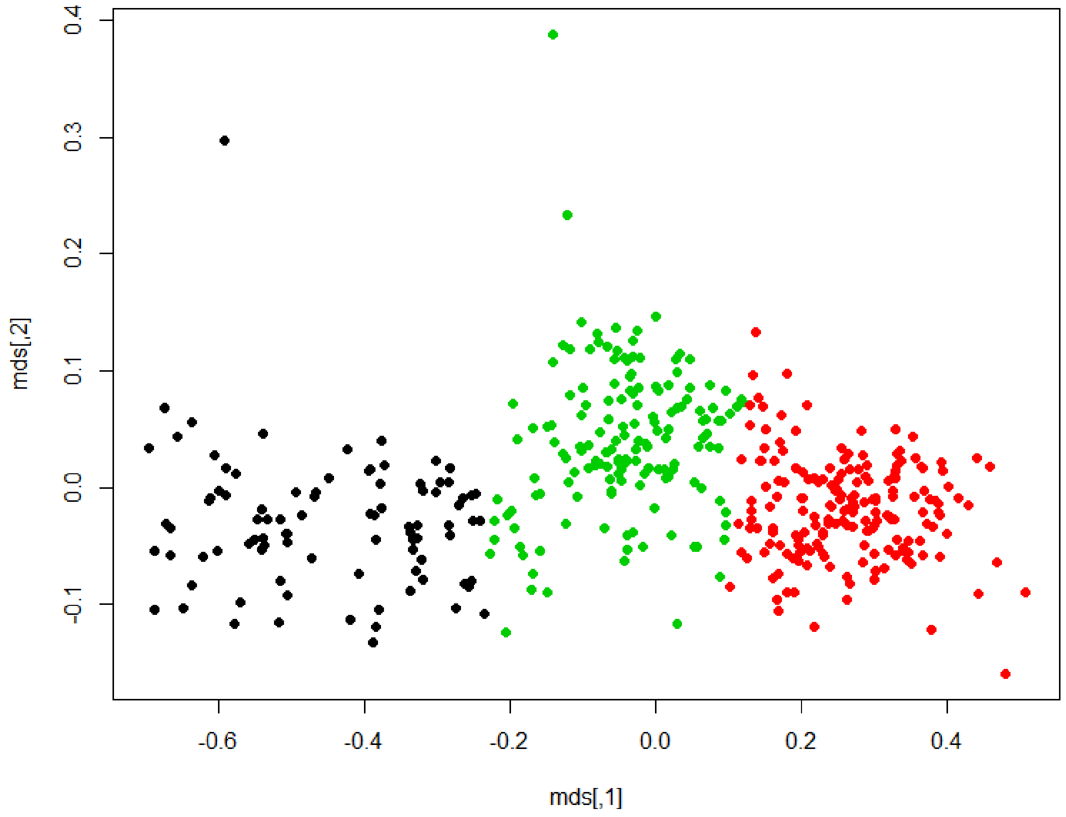

Figure S9. Clustering results of uterine corpus endometrial carcinoma samples by K-means algorithm (K = 3, after MSD conversion)

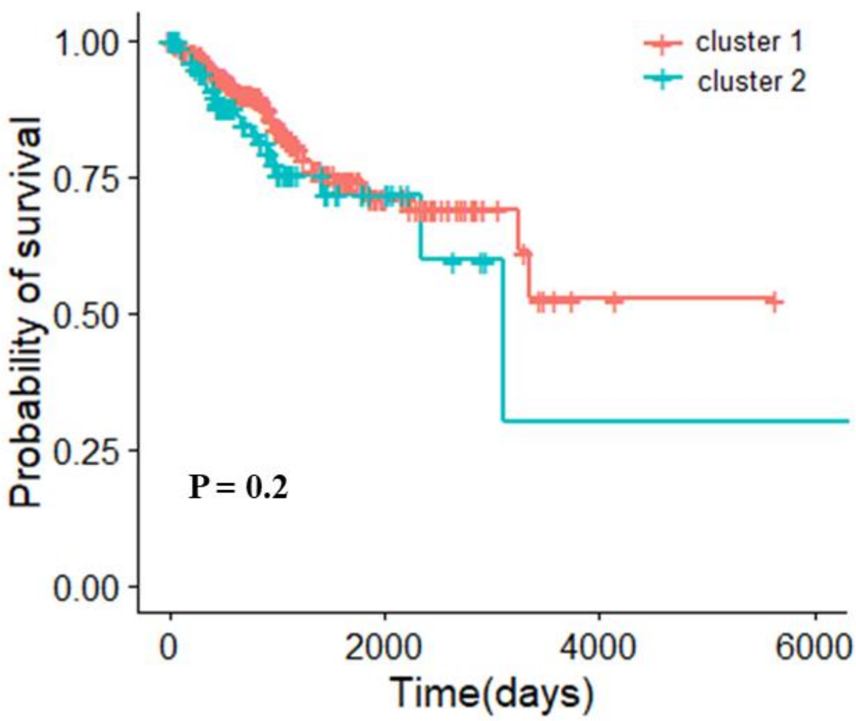

Figure S10. Survival analysis of different types of uterine corpus endometrial carcinoma samples after clustering (K = 2)

Tables

Table S1. The detailed results of preprocessing

Cancer type	Number of samples	Number of genes after expression data preprocessing	Number of genes after methylation data preprocessing
Breast invasive carcinoma	780	18,955	16,850
Skin cutaneous melanoma	468	18,955	17,031
Uterine corpus endometrial carcinoma	428	18,955	17,081

Table S2. The list of genes included in the core modules of the breast invasive carcinoma

Module Number	Number of genes included in the module	Genes contained in the module
68	6	AGBL5; CCT7; TBL3; RAB11B; PFKM; SNX5;
118	40	CRTAP; FAM113A; TYW1; FBXO31; ACCN2; B9D1; RCHY1; SSBP3; AFG3L2; RPP38; AP1B1; C21orf66; RFC2; C18orf21; TRIM25; PAICS; C16orf62; STAG1; ORC3L; C7orf46; CIAO1; NCAPG2; CEP63; CTXN1; CUL4A; DHX57; GATSL2; ZNF594; GTF3C2; SGEF; IFT20; KDM1B; KIAA0494; MGC57346; NDUFC1; NAAA; SOS1; STRA13; UQCRFS1; WDR91;

Table S3. The list of genes included in the core modules of the skin cutaneous melanoma

Module Number	Number of genes included in the module	Genes contained in the module
73	15	ABCF1; LOC440926; UBE2N; GLI4; MADD; EEF2K; ANKRD50; APPL1; API5; TSG101; C1orf122; CTSD; ZNF580; PHF12; TBK1;
98	11	SEPHS2; RBM16; BCL2L2; PDZRN3; MMP14; C21orf59; RHOJ; PROX1; SAMD5; LARGE; MPST;
122	15	CCDC9; NR1H3; SPOPL; TRIM3; C17orf63; CCDC12; MIR572; PLCD4; PRELP; MIA; OTUB1; CARS; NAT14; TET3; VMO1;
123	57	LAMC3; ABCC3; KCNJ5; SLC25A13; HCG11; ADAMTSL4; SDC1; ADIPOR1; CISH; PLK1S1; SLC25A35; YTHDF1; MAPK6; UGDH; UEVLD; TRAF4; C16orf5; FTO; OTUD1; RYBP; XKR9; FMNL2; C8orf45; PCGF6; C8orf73; C9orf3; MIR24-1; MIR23B; PHF2; CD9; IGFBP4; TMEM14A; HAP1; CREG1; CYB561; MAGI1; EFNA1; EPOR; EXT1; ETFA; PPP3CA; MEX3A; POLD2; MED24; PVRL3; TACR1; ITGA5; KIF18B; LSM11; MAGI2; PLOD1; PCBD2; PIGX; RGS9; RNF26; TSTD2; VAMP2;

Table S4. The list of genes included in the core modules of the uterine corpus endometrial carcinoma

Module Number	Number of genes included in the module	Genes contained in the module
37	8	JMJD6; SETD3; POP1; RWDD2A; NDUFS3; CDH24; GCSH; HELLS;
68	78	UBA5; AKAP5; DMXL2; FBXW7; AURKA; PSMB9; LIN37; FAM13B; ARF1; ALS2CR8; CCDC148; HELQ; PPP2R5E; CISD1; MIR636; ZNF808; ANO1; ARSJ; TBL3; ARFGAP1; STYX; ARHGDIA; ATP5I; RPS5; C17orf90; MNT; ILF3; NEK11; C17orf48; BAG2; UBE2F; PGD; C20orf43; SF3B2; C2orf76; ZNF148; MIR760; ZNF211; CAMSAP1L1; CARS2; DOCK4; WRB; ZNF862; LOC338758; CHTF18; EZH2; L2HGDH; PRKAG1; CIB2; UBE2L6; NIP7; DDX41; DDX46; SRPR; FAM134A; FGFR1OP2; WAPAL; FRAT2; HNRNPC; GGNBP2; GMDS; GTF2I; HAUS6; JAG1; PCM1; KIAA1377; LAMP3; LOC401431; ZNF598; Magmas; POLH; RFPL2; SAP30BP; SEMA3A; TDRD7; ZNF181; WDR90; ZCCHC17;
